# Acute kidney injury after trauma: Prevalence, clinical characteristics and RIFLE classification

**DOI:** 10.4103/0972-5229.74170

**Published:** 2010

**Authors:** Krasnalhia Lívia S. de Abreu, Geraldo B. Silva Júnior, Adller G. C. Barreto, Fernanda M. Melo, Bárbara B. Oliveira, Rosa M. S. Mota, Natália A. Rocha, Sônia L. Silva, Sônia M. H. A. Araújo, Elizabeth F. Daher

**Affiliations:** **From:**1 Division of Nephrology, Department of Internal Medicine, School of Medicine, Walter Cantídio University Hospital, Ceará, Brazil; 2 Department of Statistics, Science Center, Federal University of Ceará – UFC, Ceará, Brazil; 3 Department of Internal Medicine, School of Medicine, University of Fortaleza – UNIFOR, Fortaleza, Ceará, Brazil

**Keywords:** Acute kidney injury, mortality, outcome, risk factors, trauma

## Abstract

**Background:**

Acute kidney injury (AKI) is an uncommon but serious complication after trauma. The objective of this study was to evaluate the prevalence, clinical characteristics and outcome of AKI after trauma.

**Patients and Methods:**

This was a retrospective study performed from January 2006 to January 2008 in an emergency specialized hospital in Fortaleza city, northeast of Brazil. All patients with AKI admitted in the study period were included. Prevalence of AKI, clinical characteristics and outcome were investigated.

**Results:**

Of the 129 patients admitted to the intensive care unit (ICU), 52 had AKI. The mean age was 30.1 ± 19.2 years, and 79.8% were males. The main causes of AKI were sepsis in 27 cases (52%) and hypotension in 18 (34%). Oliguria was observed in 33 cases (63%). Dialysis was required for 19 patients (36.5%). Independent risk factors associated with AKI were abdominal trauma [odds ratio (OR) = 3.66, *P* = 0.027] and use of furosemide (OR = 4.10, *P* = 0.026). Patients were classified according to RIFLE criteria as Risk in 12 cases (23%), Injury in 13 (25%), Failure in 24 (46%), Loss in 1 (2%) and End-stage in 2 (4%). Overall in-hospital mortality was 95.3%. The main cause of death was sepsis (24%). Mortality was 100% among patients with AKI.

**Conclusions:**

AKI is a fatal complication after trauma, which presented with a high mortality in the studied population. A better comprehension of factors associated with death in trauma-associated AKI is important, and more effective measures of prevention and treatment of AKI in this population are urgently needed.

## Introduction

Acute kidney injury (AKI) is a complex disorder common in critically ill patients, which has been reported to affect from 1 to 25% of intensive care unit (ICU) patients and has led to mortality rates ranging from 15 to 60%.[1–4] AKI is an uncommon but serious complication after trauma. In large trauma populations, the incidence of post-traumatic AKI varies from 0.098 to 8.4% in published series[5,6] with mortality ranging from 7 to 83%.[5,7–9]

The natural history of post-traumatic AKI is not well established. Recent publications cite decreased renal perfusion as the common cause of this complication,[10,11] while the early literature suggested that AKI was secondary to crush injuries and rhabdomyolysis.[12] Renal abnormality observed is death or damage of tubular cells due to the imbalance between oxygen supply and demand of energy by hypoperfusion.[13–15]

Improvements in treatment, including the introduction of dialysis, have not changed the mortality rates of AKI.[16] The aim of this study was to investigate the prevalence, clinical manifestations and outcome of AKI in patients admitted to an ICU of trauma.

## Patients and Methods

This was a retrospective study performed from January 2006 to January 2008 with patients consecutively admitted to the ICU of a trauma specialized hospital in Fortaleza city, northeast of Brazil. Data were collected from medical record review of trauma registry. Demographic characteristics and specific information, such as cause of AKI, co-morbidities presented by each patient, use of medications, time to develop AKI after ICU admission, length of hospital stay, need for surgery, mechanism of injury, time to beginning dialysis and the cause of death, were evaluated. All clinical signs and symptoms presented by each patient at hospital admission and laboratory data during hospital stay were analyzed.

The protocol of this study was approved by the ethical committee of the Dr. José Frota Institute.

AKI was defined according to the RIFLE criteria, based on creatinine, and patients were investigated for the presence of AKI during the hospital stay.[1] Hypotension was defined as mean arterial blood pressure (MAP) of <60 mmHg and therapy with vasoactive drugs was initiated when MAP remained lower than 60 mmHg. Systolic blood pressure (SBP) and diastolic blood pressure (DBP) at admission were also analyzed. Sepsis was defined according to the American College of Chest Physicians/Society of Critical Care Medicine (ACCP/SCCM) as “the systemic response to infection, manifested by two or more of the following conditions as a result of infection: (1) temperature > 38°C or <36°C; (2) heart rate > 90 beats/minute; (3) respiratory rate > 20 breaths/minute or PaCO_2_ < 32 mmHg; and white blood count > 2000/mm^2^, <4000/mm^2^ or >10% immature (band) forms”.[17] Hypovolemic shock was differentiated from septic shock when a patient without sepsis, i.e., those who did not fill the criteria for sepsis by ACCP/SCCM developed hypotension. Metabolic acidosis was defined as pH of <7.35 and arterial bicarbonate of <20 mEq/L; and coagulation abnormalities were defined a platelet count of <100 × 10^3^ /mm ^3^. Oliguria was considered to be present when the urinary volume was less than 400 mL/day despite appropriate fluid replacement. Rhabdomyolysis was defined as creatine kinase (CK) level of >1000 IU/L. Other laboratory data evaluated were total blood count, aspartate aminotransaminase (AST), and alanine aminotransaminase (ALT). Renal-specific variables collected included admission, peak and discharge creatinine, levels of urea, potassium, sodium, and the main signs and symptoms at AKI diagnosis. Outcomes included length of stay in the ICU and hospital, use of antibiotics, need for dialysis and mortality.

RIFLE defines three grades of increasing severity of AKI – risk (class R), injury (class I) and failure (class F) – and two outcomes (Loss and End-stage kidney disease).[18] We used the change in the serum creatinine level to classify patients according to the RIFLE criteria. Patients who met any of the criteria of RIFLE classification were classified as AKI.

Patients were divided into four groups: patients with and without AKI, renal replacement therapy (RRT) and non-renal replacement therapy. We compared these groups in order to investigate the differences in clinical manifestations and laboratory features. Risk factors associated with AKI were investigated through a univariate and multivariate analysis.

The results were expressed through tables and summary measures (mean ± standard deviation) in the cases of quantitative variables. Data were analyzed with SPSS version 10.0 (SPSS Inc., Chicago, IL, USA) and Epi Info version 6.04b (Centers for Disease Control and Prevention) software. Comparison of parameters of the four groups (patients with and without AKI, RRT and non-RRT) was done with Student’s *t*-test and Fischer’s exact test. A logistic regression model was used for quantitative variables. Adjusted odds ratios (ORs) and 95% confidence intervals (CIs) were calculated. A multivariate logistic regression was performed to analyze the possible risk factors for AKI. The factors included in the multivariate model were those that showed a significance level <10% in the univariate analysis (Mann–Whitney test and chi-square test). *P* values <0.05 were statistically significant.

## Results

During the study period, 129 trauma patients were admitted to the ICU. The mean age was 30 ± 19 years (32 years for men, range 0–89 years; and 22 years for women, range 0–67 years) (*P* = 0.016); 103 (79.8%) patients were males. A total of 10 children were included, with age from 0 to 13 years. The main cause of admission was brain trauma (70.5%), followed by abdominal trauma (13.1%), thoracic trauma (10.8%) and bone fractures (37.9%). The main causes of these traumas observed were motorcycle accident (29.5%), trampling (25.6%), falls (16.2%), car accident (14%) and aggression (10.1%). Surgical procedure was required for 55 patients (42.6%), with 29.1% requiring laparotomy, 23.6% craniotomy, 21.8% orthopedic surgery, 20% thoracotomy and 5.5% others. Co-morbidities were observed in 19 (14.7%) patients; 7 (5.4%) of them had diabetes mellitus, 5 (3.9%) had hypertension and 3 (2.3%) had previous stroke. Within this population, 52 patients (40.3%) had AKI. The mean age of patients in AKI group was 32 ± 19 years (12–51 years) and 84.6% were males. The mean length of ICU stay was 31 ± 89 days (28 ± 104 days for the patients with AKI, range 2–92 days; and 33 ± 78 days for the patients without AKI, range 2–112 days). The time between ICU admission and the development of AKI was 4 ± 5 days. All the patients required mechanical ventilation, and hydration with saline solution was supplied for all cases.

The admission serum creatinine in the AKI group was 0.8 ± 0.2 mg/dL and 14 (26.9%) patients had AKI at admission. The mean laboratory data presented at non-RRT: The RRT group in comparison with non-RRT group diagnosis were: creatinine 2.7 ± 1.7 mg/dL (0.9–9.0 mg/dL), urea 86 ± 44 mg/dL (24–255 mg/dL), with 4 (7.69%) patients presenting the following: urea > 150 mg/dL; sodium 148 ± 15 mEq/L (123–143 mEq/L), potassium 4.9 ± 1.4 mEq/L (1.8–6.3 mEq/L), hemoglobin 9.9 ± 2.9 g/dL (3.3–17.1 g/dL). Twenty-seven (51.9%) patients showed the following: hemoglobin < 10 g/dL; hematocrit 30 ± 8.9% (11.3–51.6%), white blood count 19,844 ± 32,955/mm^3^ (13,500–24,660/mm^3^), platelets 205,326 ± 121,017/mm^3^ (35,000–724,000/mm^3^). Ten (19.2%) patients had platelets <100 × 10^3^/mm^3^, arterial pH 7.23 ± 0.14 (6.86–7.5), HCO_3_ 17 ± 4.1 mEq/L (9.0–28 mEq/L). Eighteen (69.2%) of 26 patients had HCO_3_ level <20 mEq/L, pCO_2_ of 42 ± 19 mmHg (30–57 mmHg), and pO_2_ of 123 ± 72 mmHg (34–101 mmHg). Eleven (47.8%) of 23 patients had the following: pO_2_ < 90 mmHg, AST 380 ± 609 IU/L (10.4–2276 IU/L), ALT 389 ± 646 IU/L (10–2,772 IU/L) and CK 3736 ± 3966 IU/L (418–13,818 IU/L). CK levels were requested in only 11 patients and rhabdomyolysis was found in 6 of them and all developed AKI. The observed prevalence of oliguria was 63% (33 cases of 52 with AKI). Among patients with AKI, 90.4% used antibiotics, 90.4% used vasoactive drugs and 9.6% used anti-inflammatory drugs.

The causes of AKI were sepsis in 27 cases (52%), hypotension in 18 (34%), acute injury in chronic renal failure in 2 (4%), drug toxicity in 2 (4%) and renal trauma in 1 (2%). Rhabdomyolysis (CK > 1000 IU/L) was found in six patients with AKI who had the CK level assessed. Sepsis was observed in 21.2% of patients with AKI and in 15.6% of those without AKI (*P* = 0.48). The frequency of hypotension was also not statistically different between AKI and non-AKI patients (5.8% vs. 15.6%, *P* = 0.10).

Independent risk factors associated with AKI were abdominal trauma (OR = 3.66, 95% CI = 1.16–11.53, *P* = 0.027) and use of furosemide (OR = 4.10, 95% CI = 1.18–14.23, *P* = 0.026). A comparison between AKI and non-AKI patients is summarized in Table 1. Furosemide was administered for 14 patients (10.8%), and all patients who had received the medication died.

**Table 1:** Comparison of patients with and without acute kidney injury after trauma

Parameter	AKI (n = 52)	Non-AKI (n = 77)	OR	95% CI	P
Age (years)	31 ± 19	28 ± 19	—	—	0.29
Gender			—	—
Male	44 (84.6)	59 (76.6)			0.37
Female	8 (15.4)	18 (23.4)			
Type of trauma					
Brain	38 (73.1)	67 (87)	3.86	1.25–11.89	0.06
Abdominal	11 (21.2)	5 (6.5)			0.02
Polytraumatism	4 (7.7)	13 (16.9)			0.18
Co-morbidities			—	—	
Diabetes mellitus	3 (5.8)	4 (5.2)			1.0
Hypertension	2 (3.8)	3 (3.9)			1.0
Stroke	2 (3.8)	1 (1.3)			0.56
Medications at admission					
Furosemide	10 (19.2)	4 (5.2)	4.34	1.28–14.7	0.01
Vancomycin	6 (11.5)	6 (7.8)			0.54
Cefepime	10 (19.2)	11 (14.3)			0.45
Vasoactive drugs	7 (13.4)	10 (13)			0.26
Death	51 (98.1)	72 (93.5)	—	—	0.40

OR, odds ratio; CI, confidential interval, Values given in the parenthesis are in percentage

Of the 52 patients who developed AKI, 19 required RRT. This group represented 36.5% of patients with AKI and 14.7% of all patients admitted at ICU. This group consisted of 100% of men whose average age is 38 ± 14 years (21–66 years). A comparison between patients who required RRT and those who did not require is summarized in Table 2.

**Table 2:** Comparison of patients with acute kidney injury after trauma, who required renal replacement therapy with those who did not require renal replacement therapy

Parameter	RRT (n = 19)	Non-RRT (n = 33)	P
Age (years)	38 ± 14	28 ± 20	<0.0001
Gender			
Male	19 (100)	25 (75.7)	0.05
Female	0	8 (24.3)	—
Length of ICU stay (days)	16 ± 20	6 ± 5	0.002
Systolic blood pressure (mmHg)	100 ± 42	98 ± 35	0.26
Diastolic blood pressure (mmHg)	57 ± 18	58 ± 21	0.43
Time to develop AKI after ICU admission (days)	6 ± 8	3 ± 2	0.02
Signs and symptoms			
Uremia	19 (100)	24 (72.7)	0.61
Sepsis	10 (52.6)	19 (57.5)	0.72
Hyperkalemia	11 (57.8)	9 (27.2)	0.02
Metabolic acidosis	9 (47.3)	15 (45.4)	0.89
Hypotension	10 (52.6)	15 (45.4)	0.61
Oliguria	9 (47.3)	18 (54.5)	0.61
Laboratory data			
Creatinine at admission (mg/dL)	0.9 ± 0.3	0.7 ± 0.2	0.02
Creatinine in AKI diagnosis (mg/dL)	3.9 ± 2.1	2.0 ± 0.9	0.03
Maximum creatinine (mg/dL)	5.5 ± 3.3	2.3 ± 1.0	<0.0001
Creatinine before death (mg/dL)	3.3 ± 1.8	1.9 ± 0.9	<0.0001
Urea at admission (mg/dL)	45 ± 18	43 ± 24	0.14
Urea in AKI diagnosis (mg/dL)	101 ± 55	77 ± 34	<0.0001
Maximum urea (mg/dL)	169 ± 70	95 ± 49	<0.0001
Urea before death (mg/dL)	124 ± 68	89 ± 55	<0.0001
Potassium in AKI diagnosis (mEq/L)	5.5 ± 1.2	4.6 ± 1.5	0.01
CK in AKI diagnosis (IU/L)*	2313 ± 1401	2906 ± 2350	0.45
Arterial pH in AKI diagnosis	7.22 ± 0.10	7.23 ± 0.16	0.92
HCO_3_ in AKI diagnosis (mEq/L)	17.7 ± 2.16	17.5 ± 5.0	0.72
RIFLE classification			
Risk	0	12 (36.3)	—
Injury	2 (10.6)	11 (33.3)	0.13
Failure	14 (73.6)	10 (30.4)	0.02
Loss	1 (5.2)	0	—
End-stage	2 (10.6)	0	—
Renal function recovery before death	2 (10.5)	11 (33.3)	0.13
Death	18 (94.7)	33 (100)	—

CU, intensive care unit, Mean ± SD; significant when *P* < 0.05, *CK in AKI diagnosis (RRT, *n* = 3; non-RRT, *n* = 7), , Values given in the parenthesis are in percentage

**Figure 1:**
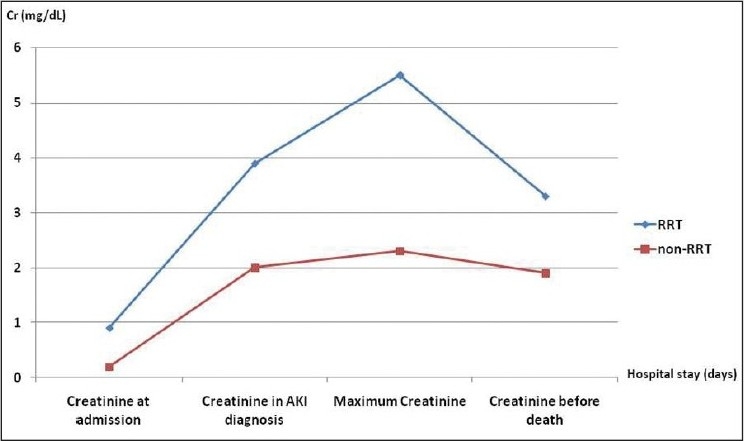
Comparison of mean serum creatinine in different occasions in patients with acute kidney injury after trauma, who required renal replacement therapy and those who did not require (non-RRT)

**Figure 2:**
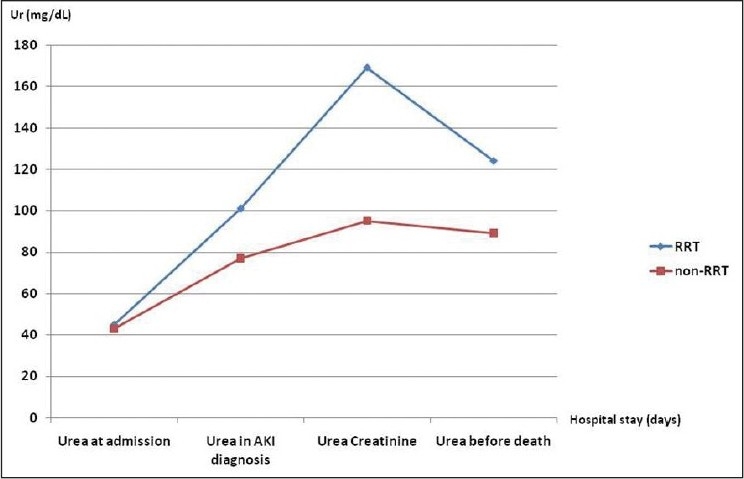
Comparison of mean serum urea in different occasions in patients with acute kidney injury after trauma, who required renal replacement therapy and those who did not require

The RRT group in comparison with non-RRT group had higher creatinine level at admission (0.9 ± 0.3 mg/dL vs. 0.7 ± 0.2 mg/dL, respectively, *P* = 0.02) and higher maximum creatinine level (5.5 ± 3.2 mg/dL vs. 2.3 ± 1.0 mg/dL, respectively, *P* < 0.0001), as illustrated in Figure 1. In addition, the RRT group presented AKI later (6 ± 8 days vs. 3 ± 2 days, respectively, *P* = 0.02). The RRT group had higher peak serum urea (169 ± 70 mg/dL vs. 95 ± 49 mg/dL, respectively, *P* < 0.0001), as illustrated in Figure 2. Patients who required RRT had a more prolonged stay in the ICU than those who did not require RRT (16 ± 20 days vs. 6 ± 5 days, respectively, *P* = 0.002).

Patients were classified according to RIFLE criteria, as Risk in 12 cases (23%), Injury in 13 (25%), Failure in 24 (46%), Loss in 1 (2%) and End-stage in 2 (4%). Overall in-hospital mortality was 95.3%. The mean cause of death was sepsis (24%). Mortality was 100% among patients with AKI. The majority of patients who required RRT were classified as Failure (74%) and patients who did not require RRT were classified as Risk (37%), Injury (33%) and Failure (30%). The causes of death were encephalic death in 61 cases (49.6%), cardiac arrest in 25 (20.3%), sepsis in 21 (17%), multiple organ dysfunction syndrome in 9 (7.3%), respiratory insufficiency in 4 (3.2%) and others in 3 (2.6%) cases. Therapy was withdrawn in patients with encephalic death.

## Discussion

The natural history of post-traumatic AKI is not well established. In retrospective studies on large trauma populations, a low incidence of AKI is generally reported (0.1–8.4%).[5,6] However, the incidence of AKI in the ICU varies from 1.5 to 24%.[2] In our population of patients admitted at an ICU, the incidence was higher (40.3%). We observed a higher prevalence of males with AKI (84% vs. 15%) because men are more frequently exposed to external injuries. Previous studies have shown similar findings but some have shown that the mortality is similar for men and women.[3,4,20,21] This study was conducted in a reference center in Brazil where accidents involving motorcycles, cars and falls are the most common causes of trauma. In larger cities, where traffic accidents and physical violence are more common, we can observe more serious injuries. It is important to note that males and younger individuals are more often involved in accidents.[21] The mean age of our patients was around 30 years, which is lower than that reported in literature (which ranges from 51 to 68 years).[4]

The causes of AKI include sepsis, hypovolemia, pre-existing renal impairment, and nephrotoxins such as aminoglycoside antibiotics and radiological contrast agents.[23,24] Causes of post-traumatic renal failure are likely to be multifactorial.[24–31] Additional risk posed by renal trauma itself must also be considered in this population, with subsequent outcomes varying because of both degree and type of injury occurring.[23] In the present study, the main causes of AKI were sepsis, hypotension and rhabdomyolysis. The low number of patients in whom CK level was assayed was responsible for only 6 patients detected with rhabdomyolysis; it probably would be higher if all patients had their CK level requested. We observed that 10 out of 11 patients with increased CK developed AKI.

In the early literature, AKI in trauma patients was reported to be mainly secondary to crush injuries and rhabdomyolysis,[12] whereas more recently decreased renal perfusion has emerged as the most common cause of AKI.[5,10,11,32,33] The reported incidence of AKI in rhabdomyolysis ranges from 13% to approximately 50% and the prognosis in these cases is substantially worse. Among patients in the ICU who developed rhabdomyolysis, the mortality has been reported to be 59% when AKI is present and 22% when it is not present.[31,34–37]

The mechanisms involved in the pathogenesis of rhabdomyolysis are direct sarcolemmic injury (e.g., trauma) or depletion of ATP within the myocyte, leading to an unregulated increase in intracellular calcium.[38,39] In the case of patients with rhabdomyolysis caused by trauma, additional injury results from ischemia reperfusion and inflammation by neutrophils that infiltrate damage muscle.[40]

The value of CK above which the risk of AKI is significantly higher is not set. AKI may be related to values as low as 500 IU/L, but this usually occurs when there are conditions such as sepsis, dehydration and metabolic acidosis,[31] similar to that observed in our study.

Brivet *et al*.,[4] in a prospective study from 282 patients with AKI in an ICU, found sepsis in 48%, hemodynamic dysfunction (excluding sepsis) in 32% and toxic injuries in 20%, similar to our results. Sepsis is one of the main causes of AKI in ICUs and is associated with worse prognosis.[3,41–46] Patients with sepsis have generalized arterial vasodilatation, with an associated decrease in renal vascular resistance, which causes renal hypoperfusion and AKI.[45]

Regel *et al*.[6] showed that during recent decades, the dramatic increase in intravenous fluid administration to trauma patients in the first 24 hours after injury has markedly reduced the incidence of AKI and has improved the outcome.

The major findings of the present study were that abdominal trauma and use of furosemide were independent risk factors for AKI in our population. During trauma, the consequences of elevated intra-abdominal pressure are also significant determinants for the impairment of renal function. However, experimental studies show that the impairment in renal function produced by increased intra-abdominal pressure is a local phenomenon caused by direct renal compression and is not related to cardiac output.[47]

Furosemide is frequently used to facilitate fluid and electrolyte management of AKI in many institutions although its potential benefits, adverse effects, and cost effectiveness to prevent or treat AKI remain uncertain.[23] Several small, randomized, controlled studies evaluating the use of furosemide to either prevent or treat AKI have produced negative results.[22,48,49] Furthermore, the use of diuretics for AKI has also been associated with an increased risk of non-recovery of renal function and mortality.[50] Furosemide, especially at high doses, is associated with important side effects. Previous observational studies have produced conflicting results about the association between furosemide and mortality.[22,50] Indeed it is not associated with any clinical benefits when used to prevent and treat acute renal failure in adults.[22]

Efforts to establish the true incidence of AKI in trauma patients are complicated by alterations in the definition used to characterize renal dysfunction in various studies. To foster uniformity in both research and clinical practice, an expert group [Acute Dialysis Quality Initiative (ADQI)] developed a new classification of AKI that has been increasingly used, i.e., RIFLE classification. It includes both biochemical measures of renal function and urine output as components of the definition. In a meta-analysis that included patient-level data of more than 71,000 patients in 13 studies, RIFLE criteria displayed a graded association with adverse outcomes.[51,52] The overall mortality in the study was 6.9% in the group without AKI and 31.2% in the AKI group. The RIFLE classification was associated with increased risk of death and decreased likelihood of renal recovery. The majority of patients included in the present study were classified as “Failure”, according to RIFLE classification. RIFLE was not an independent predictor of mortality in this ICU population with post-traumatic AKI, which may be due to the high mortality rate (almost all patients died, so it was not possible to find specific risk factors for death). This fact may be due to the specific clinical conditions and severity of the trauma that override renal lesions in determining mortality. The majority of patients in the present study who required RRT were in “Failure” stage, but a high proportion of patients in this stage did not received RRT, which may be due to the severity of disease (most patients died before RRT could be initiated). In a recent study by Bagshaw *et al*.[53] with 9449 critically ill trauma patients, AKI was found in 18.1%, which was lower than that observed in the present study, and the factors associated with AKI were older age, female gender and the presence of co-morbidities. Another recent study with 3945 patients admitted to a Chinese hospital after road traffic injury found AKI in a lower proportion of cases (10.7%), and the risk factors for AKI were use of vasopressor drugs for more than 4 hours, use of high-dose diuretics and delayed transport time.[54]

AKI is associated with a significant risk of morbidity and mortality (rates as high as 78% in patients who require RRT).[22] In our study, the overall in-hospital mortality was 95.3% and it was 100% among patients with AKI. Both RRT and non-RRT group had no difference in mortality, despite differing in severity of renal failure. Gomes *et al*.,[55] in a recent study with 436 trauma patients admitted to an ICU in Portugal, observed AKI in 50% of cases. Overall mortality was 30%, which was significantly lower than that observed in the present study. Late mortality (2 days after admission) was 18% among AKI patients and 22% in non-AKI (*P* = 0.31) patients, showing that AKI was not associated with increased mortality in trauma patients, as observed in our study. Bagshaw *et al*.,[53] however, found a significantly higher mortality among trauma patients with AKI (16.7% vs. 7.8%, *P* < 0.001). Yuan *et al*.[54] also found higher mortality in patients with AKI, and this was higher according to the RIFLE criteria (mortality was 7.1% in patients without AKI, 37.4% in Risk, 52.9% in Injury and 79.2% in Failure). The mortality rate in our study was very high, but we do not know the actual reason for this. We believe that this was due to the severity of studied cases. The majority of patients had severe AKI (classified as “Injury” of “Failure”), and this is associated with high mortality. Another possible reason for the high mortality could be related to a delay in the beginning of dialysis therapy, due to technical problems.

Because of the high mortality rates, prevention of AKI in severe trauma patients admitted to the ICU remains crucial. The risk factors for post-traumatic AKI identified in the present study may help the provision of future strategies. The majority of patients were young males, and the high mortality is surprising since mortality was expected to be lower in this group of patients. We can attribute this to the fact that the patients were victims of severe trauma, which is very common in the hospital where they were admitted.

### 

#### Limitations of the Study

The study limitations were its small population, insufficient resource to allow complete data collection and limitation to the required laboratory data. The small sample size is probably an important factor for not being able to show any effect of AKI on mortality even if our mortality rates had been different. We believe, however, that our results provide some information that will be useful in clinical practice.

## Conclusion

The prevalence of AKI and overall mortality of our patients was higher than that reported in the literature. AKI is a frequent and fatal complication after trauma. RIFLE classification was not a predictor factor for mortality in our ICU post-traumatic AKI population. A better comprehension of risk factors associated with death in patients with trauma-associated AKI is important, and more effective measures of prevention and treatment of AKI in this population are urgently needed.
